# The Dynamics of Seizures After Microsurgical Treatment of Brain AVMs in Patients with Symptomatic Epilepsy: A Single-Center Experience over 10 Years

**DOI:** 10.3390/medicina61050856

**Published:** 2025-05-06

**Authors:** Yerbol Makhambetov, Aiman Maidan, Iroda Mammadinova, Karashash Menlibayeva, Baurzhan Kunakbayev, Serik Dyussembaev, Nurtay Nurakay, Nursultan Makhambetov, Aigul Almabayeva, Chingiz Nurimanov

**Affiliations:** 1 National Centre for Neurosurgery, Astana 010000, Kazakhstan; yermakh@gmail.com (Y.M.); irodamammadinova@gmail.com (I.M.); kara.mnl.kz@gmail.com (K.M.); kunakbayev@gmail.com (B.K.); dr.serik.d@gmail.com (S.D.); nurtaynurakay92kz@gmail.com (N.N.); nmahambetov7@gmail.com (N.M.); chingiz198705@gmail.com (C.N.); 2 Taraz City Multidisciplinary Hospital and Consulting and Diagnostic Center, Taraz 080000, Kazakhstan; 3 Anatomy Faculty, Astana Medical University, Astana 010000, Kazakhstan; topaika@mail.ru

**Keywords:** arteriovenous malformation, structural epilepsy, ILAE classification, Engel classification

## Abstract

*Background and Objectives*: Arteriovenous malformations (AVMs) are abnormal connections between arteries and veins, lacking a normal capillary network. Seizures are a common clinical manifestation in patients with brain AVMs, ranking as the second most frequent presentation. The objective of this study was to evaluate the dynamics of seizure activity in patients with brain AVMs following surgical treatment. *Materials and Methods*: This study included 27 patients with brain AVMs who underwent microsurgical AVM resection for symptomatic epilepsy. All surgical interventions were performed at JSC “National Centre for Neurosurgery” between 2008 and 2020. *Results*: Over an average follow-up period of 98.07 ± 45.6 months, 82 patients with brain AVMs underwent open microsurgical resection at the National Centre for Neurosurgery. Among them, 27 patients presented with seizures and had complete follow-up information, qualifying them for inclusion in this study. The participants had a mean age of 32.59 ± 9.06 years, with 13 of them being women. The Spetzler–Martin grading system was used to classify the AVMs: 6 patients had grade 1, 13 had grade 2, 7 had grade 3, and 1 had grade 4. More than half of the patients experienced generalized seizures. Microsurgical removal of the AVMs resulted in seizure remission for all patients. Only one patient experienced postoperative hemorrhage during the follow-up period. Additionally, one patient developed acute postoperative anemia, which resolved with a favorable outcome. *Conclusions*: Microsurgical resection of brain AVMs, when performed with careful patient selection, leads to a significant reduction in seizure activity. It is a safe and effective treatment option for symptomatic epilepsy associated with brain AVMs.

## 1. Introduction

Cerebral arteriovenous malformations are pathological vascular lesions characterized by a network of aberrant arteries and veins lacking a complete capillary bed [1]. This type of vascular anomaly is a common etiology of intracranial hemorrhage, especially in younger populations [2]. Seizures represent the second most frequent clinical manifestation in patients with cerebral AVMs [3]. While the precise mechanisms underlying AVM-associated seizures remain incompletely understood, various factors have been proposed, including reduced perfusion of surrounding brain tissue, scarring, neuronal injury, hemosiderin deposition from prior hemorrhage, and heightened neural excitability. The treatment of these lesions through microsurgical resection is well established, but the impact on long-term seizure control remains an important clinical concern [4].

Research indicates that a significant proportion of patients with cerebral arteriovenous malformations, ranging from 12% to 57%, experience seizures, which can persist even after surgical intervention [5]. Uncontrolled seizures can lead to severe complications and substantially diminish the quality of life [6]. However, the issue of managing symptomatic epilepsy in these patients is often overlooked, as the primary focus of research has been on the risk of hemorrhage associated with AVMs rather than the problem of structural epilepsy [7]. The limited understanding of the underlying causes of seizures and the factors influencing seizure remission after surgical treatment is attributed to the heterogeneity of clinical presentations and the paucity of research in this area [8]. While seizures are a frequent clinical presentation in patients with AVMs, the degree of treatment resistance varies across individuals. In this study, treatment-refractory epilepsy was defined as the persistence of seizures despite adequate trials of two or more antiepileptic medications. Due to resource limitations, intraoperative electrocorticography (ECoG) was not routinely employed in our surgical protocol, as detailed in the Methods and Limitations sections. Clarifying this distinction is crucial for understanding the characteristics of our patient cohort, the surgical strategy applied, and the seizure outcomes observed.

Although the existing literature highlights favorable seizure outcomes following microsurgical AVM resection, no such data currently exist for the Kazakh population. This retrospective study addresses that gap by examining long-term seizure outcomes in patients with AVM-associated, treatment-resistant epilepsy who underwent microsurgical intervention at a single national neurosurgical center over a 10-year period.

## 2. Materials and Methods

### 2.1. Study Design

This was a retrospective observational study that included patients over 18 years old with cerebral arteriovenous malformations and symptomatic epilepsy who underwent microsurgical resection at the National Research Center between 2008 and 2020. The researchers analyzed variables such as the type and nature of seizures, the use of antiepileptic drugs before and after treatment, and potential side effects. The collected data encompassed patient demographics, clinical characteristics, details of the surgical intervention, neuroimaging findings, AVM features, and treatment outcomes. Particular emphasis was placed on the history of seizures, including onset, type, and effectiveness of drug management. Epilepsy was considered treatment resistant when seizures persisted despite the use of more than two antiepileptic medications. All participants underwent preoperative neuroimaging, including cerebral angiography, to document AVM size, location, and involvement of functionally significant brain areas. The AVMs were also evaluated using the Spetzler–Martin and Lawton–Young scales, and the history of hemorrhage and previous embolization was noted.

### 2.2. Sample Size

**Figure 1:**
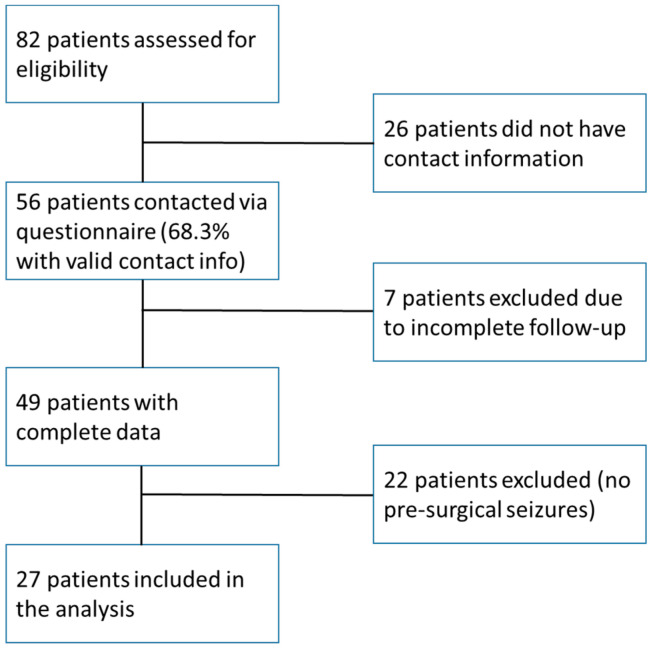
Inclusion and exclusion flowchart.

Over the follow-up period, 82 patients who had undergone microsurgical resection of brain AVMs were screened for eligibility (Figure 1). Of these, 56 patients (68.3%) had valid contact details and were successfully reached via questionnaire. Further, 7 individuals were excluded due to incomplete follow-up, leaving 49 patients with full postoperative data. Among them, 22 had no history of seizures prior to surgery and were excluded, resulting in a final study cohort of 27 patients. Treatment-resistant epilepsy was defined as continued seizure activity despite the use of two or more appropriate and tolerated antiepileptic drugs. All included patients fulfilled this criterion. Those lacking postoperative seizure outcome data or with follow-up shorter than 12 months were excluded, a potential source of selection bias, as acknowledged in the Limitations section. Eligibility criteria extended beyond seizure history and included age over 18, imaging-confirmed AVM diagnosis, and microsurgical treatment with curative intent. Patients with neurocutaneous disorders, additional intracranial pathologies, or purely hemorrhagic presentations without epilepsy were excluded. Comprehensive data were collected for each patient, encompassing demographic details, AVM characteristics (location, grading via Spetzler–Martin and Lawton–Young systems), seizure duration and type, antiepileptic drug usage and dosages, embolization details, clinical presentation, and intra- and postoperative complications.

### 2.3. Treatment Approaches

A multidisciplinary team tailored each patient’s treatment strategy. Preoperative embolization was employed selectively, followed by microsurgical resection. Outcomes were assessed using the Engel and International League Against Epilepsy (ILAE) classification systems.

The treatment of patients with cerebral arteriovenous malformations was conducted using a multifaceted approach, which involved open surgery and embolization. This comprehensive treatment plan was developed through careful discussion by a multidisciplinary team comprising neurosurgeons with dual expertise and neurologists specializing in epilepsy. The surgical interventions were performed using microsurgical techniques that aimed to maximize the removal of AVMs while minimizing damage to the surrounding brain tissue. Due to technical limitations at our institution during the study period, intraoperative electrocorticography (ECoG) was not routinely employed in the surgical management of AVM-associated epilepsy. However, microsurgical resection was guided by anatomical landmarks and preoperative imaging, including angiography and MRI, to ensure complete nidus excision.

In some cases, embolization of the feeding vessels was performed prior to the subsequent microsurgical resection.

### 2.4. Primary and Secondary Outcomes

Postoperative outcomes were assessed using a modified Engel classification, grouping patients into two categories: those who were seizure free or experienced only a single seizure and those with recurrent seizures at the final follow-up. To enhance the granularity of outcome evaluation, the International League Against Epilepsy (ILAE) outcome scale was also applied. The primary endpoint was the seizure-free rate at 1, 3, and 5 years following the microsurgical intervention. Secondary outcomes included the proportion of patients with significant seizure reduction (defined as a >50% decrease in seizure frequency), adjustments in antiepileptic drug regimens, and patient-reported quality-of-life improvements. Kaplan–Meier survival analysis was used to estimate seizure-free survival over time. Engel and ILAE classifications were applied using established criteria—Engel IA indicated complete seizure freedom and ILAE Class 1 represented no postoperative seizures. Inter-rater reliability was maintained through independent evaluations by two clinicians, with discrepancies resolved by a senior neurologist. Recognizing the retrospective nature of this study, we addressed potential biases, including recall and selection bias, through thorough electronic chart reviews and the use of standardized outcome measures.

### 2.5. Statistical Analysis

All data were cleaned, coded, and organized using Microsoft Excel, and statistical analyses were performed using IBM SPSS Statistics version 26.0 (IBM Corp., Armonk, NY, USA). Descriptive statistics were used to summarize the dataset, including frequencies, percentages, means, and standard deviations. Fisher’s exact test was employed to evaluate associations between categorical variables due to the limited sample size and low expected cell counts. The Kaplan–Meier method was used to estimate seizure-free survival rates over time. A *p*-value of less than 0.05 was considered statistically significant.

## 3. Results

### 3.1. Patient Characteristics

**Figure 2:**
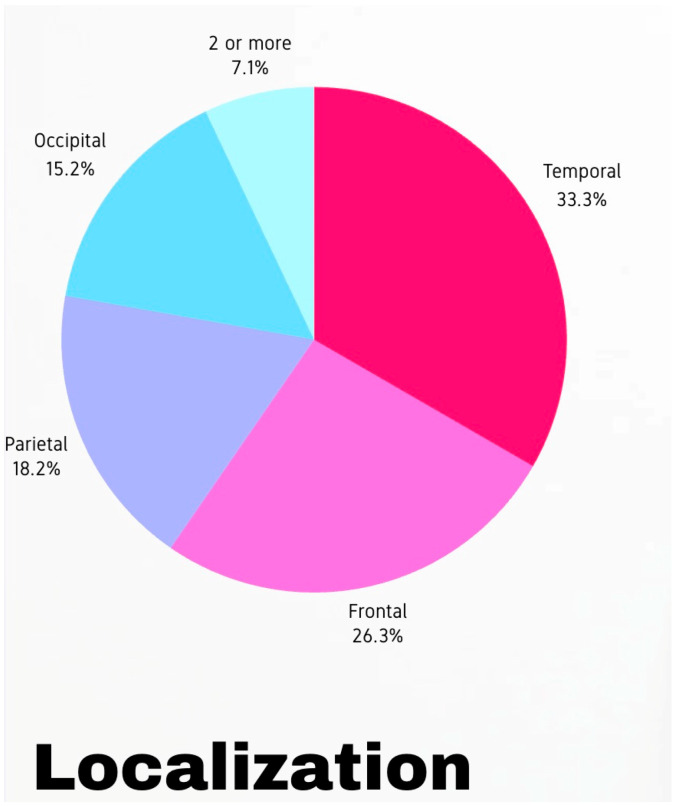
The pie chart regarding the localization.

**Figure 3:**
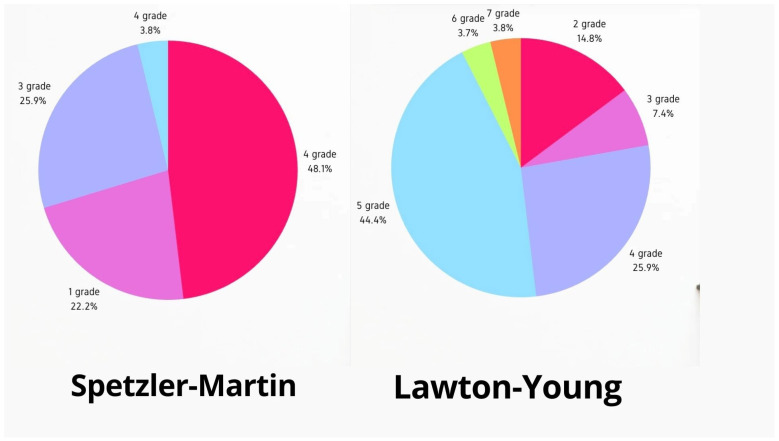
The pie charts regarding classifications.

Among 82 AVM patients treated during the study period, 27 experienced seizures and underwent microsurgical resection. The cohort’s mean age was 32.6 ± 9.08 years, and 48% were female. Seizure durations averaged 79.03 ± 98.79 months preoperatively. The AVM locations were as follows: six patients in the frontal lobe, nine in the temporal lobe, two in the frontotemporal region, five in the parietal lobe, and four in the occipital lobe (as seen in Figure 2). Left-sided AVM localization was observed in 59.2% of patients. AVM grades (Spetzler–Martin) included grade 1 (22%), grade 2 (48%), grade 3 (19%), and grade 4 (4%). According to the Lawton–Young classification, 1 patient was in grade 7, 4 in grade 6, 12 in grade 5, 6 in grade 4, 1 in grade 3, and 1 in grade 2. In the microsurgical treatment group, 48% of patients had a history of AVM rupture, and 40.7% did not require preoperative embolization. Figure 3 represents pie charts according to arteriovenous malformation classifications. Focal seizures were present in 40.7% of patients and generalized seizures in 59.3%. The general characteristics of the patients are presented in Table 1.

### 3.2. Seizure Outcomes

- 78% achieved seizure freedom (Engel IA).
- 11% experienced reduced seizure frequency (Engel IIA, IIIA).
- 11% had persistent seizures (Engel IVB).
- Generalized seizures (59%) responded more favorably than focal seizures (41%).

Following microsurgical treatment, 78% of patients achieved seizure remission. Generalized seizures were more likely to remit after surgery (Engel IA in 81% of generalized vs. 72% of focal). AVMs located in the temporal and occipital lobes showed slightly better seizure remission rates.

**Figure 4:**
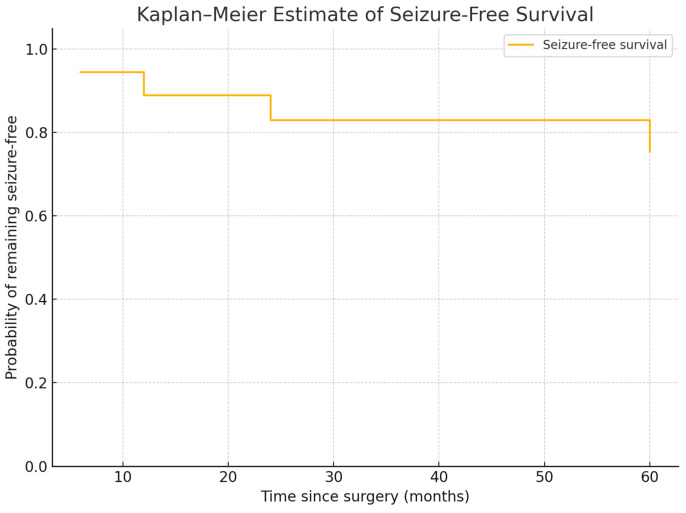
Kaplan–Meier estimate of seizure-free survival. The figure illustrates the probability of remaining seizure free following microsurgical resection over time. The analysis shows the cumulative seizure-free survival for the study cohort at defined postoperative intervals (in months).

The remaining patients were distributed as follows according to the Engel and ILAE classification systems: two patients were assigned to Engel class III, three to IIIA, and two to IVB; six patients were assigned to ILAE class 3, three to class 5, and two to class 1. Data analysis indicated that the frequency and duration of seizures prior to surgery were significant factors influencing the need for postoperative antiepileptic medication. The study results demonstrated variability in outcomes after microsurgical resection in patients with arteriovenous malformations and epilepsy, as presented in Table 2. Statistical analysis revealed significant associations between categorical clinical outcome variables following microsurgical resection. Chi-square, Likelihood Ratio, and Fisher’s exact test consistently demonstrated statistically significant correlations between postoperative seizure outcomes, as measured by Engel and ILAE classifications (*p* = 0.005). Furthermore, seizure duration prior to surgery was a significant predictor of postoperative outcomes, with longer durations associated with less favorable seizure control (*p* = 0.005). The relationship between seizure duration and postsurgical seizure outcomes is presented in Table 3 for Engel classifications and in Table 4 for ILAE classifications. These findings underscore a strong, statistically significant relationship between seizure history and surgical efficacy in AVM-related epilepsy. Seizure-free survival was estimated using the Kaplan–Meier method to illustrate the duration of postoperative seizure control (Figure 4).

**Figure 5:**
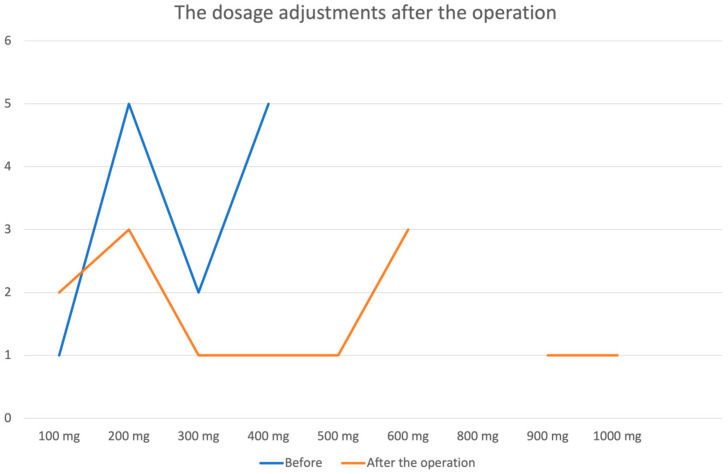
Antiseizure medication adjustments after the operation.

Among the patients who continued to experience seizures after surgery, four had not taken antiepileptic medications before the intervention but were started on carbamazepine after; two patients continued taking valproic acid, one switched from valproic acid to carbamazepine, and others increased their carbamazepine dosage, as detailed in Table 5 and Figure 5. The dosage adjustments of antiseizure medications are represented in Figure 4. Postoperative complications included one case of hemorrhage and one of anemia. Both were managed conservatively without long-term neurological deficits or worsening of seizure status.

## 4. Representative Case

**Figure 6:**
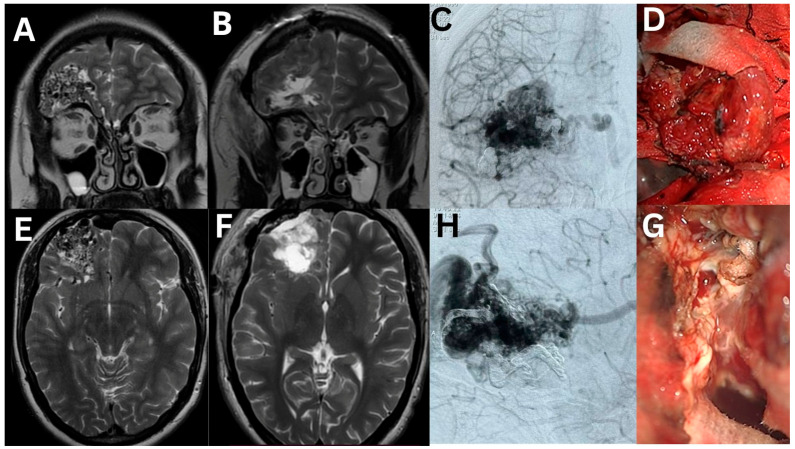
MRI, DSA, and intraoperative findings related to the arteriovenous malformation in the basal sections of the right frontal lobe. The panels include T2-weighted MRI scans (**A**,**E**), selective cerebral angiographs before and after pre-embolization (**C**,**H**), and macroscopic images intraoperatively (**G**) and after surgical excision (**D**). The postoperative T2-weighted MRI studies (**B**,**F**) demonstrate the completeness of AVM resection.

A 27-year-old female with a history of recurrent generalized tonic–clonic seizures presented with a recent convulsive episode that prompted neuroimaging and diagnosis of a right frontal AVM. Magnetic resonance imaging revealed an arteriovenous malformation in the right frontal lobe. Selective cerebral angiography visualized the AVM, which measured 20.95 × 1.67 × 4.90 mm and received afferents from the M2 segments of the right central middle cerebral artery and the A1 and A3 segments of the right anterior cerebral artery. The drainage occurred through dilated cortical veins into the anterior third of the upper sagittal sinus and the deep cerebral vein into the Galena vein. The AVM was classified as Spetzler–Martin, grade 3. A microsurgical excision of the AVM was performed after preliminary partial embolization of the afferents and the AVM nidus. MRI, angiogram, and intraoperative photos are shown in Figure 6. The postoperative period was uneventful.

## 5. Discussion

Our findings align with prior research indicating that microsurgical resection provides robust seizure control in AVM-associated epilepsy. Compared to radiosurgery and endovascular embolization, which may offer delayed or partial obliteration, microsurgery provides immediate and complete AVM removal, contributing to more favorable seizure outcomes [9]. These results are consistent with previous reports that have demonstrated the efficacy of surgical treatment in managing epilepsy associated with cerebral AVMs [10]. Factors such as the duration and frequency of seizures before surgery were found to be significant predictors of postoperative seizure control. Specifically, patients with a longer history of seizures and higher seizure frequency were less likely to achieve seizure remission after AVM microsurgery.

The use of a multimodal approach involving both embolization and microsurgical resection has been shown to enhance the effectiveness of AVM treatment [7]. In our study, 40.7% of patients did not require preoperative embolization, indicating that surgical resection alone can be an effective treatment option in some cases. Although valproate is associated with potential hematologic side effects, including thrombocytopenia, its use was limited to three patients who had no prior history of coagulopathy. Platelet counts and coagulation profiles were monitored preoperatively, and no intraoperative or postoperative bleeding complications were observed in these patients.

Arteriovenous malformations of the brain commonly present with seizures, which can significantly impair the quality of life for affected patients [11]. However, the risk factors for epilepsy in the context of AVMs have not been extensively studied, and the significance of addressing seizures in the surgical management of these lesions is often underappreciated, as most research has focused on the risk of hemorrhage [12]. In a previous publication, the authors identified factors associated with the initial development of epilepsy in their patient cohort, including the presence of hemiparesis; a Spetzler–Martin grade above III; localization in the frontal, parietal, occipital, or temporal lobe; and history of AVM rupture [13].

The present study highlights the experiences of a specialized neurosurgical center, where comprehensive diagnostic capabilities, including super-selective cerebral angiography, seizure assessment, and preoperative embolization, are available [14]. Previous publications from this center have described the impact of endovascular treatment on epilepsy management [15]. The microsurgical approach to treating AVMs that cause epileptic seizures often yields favorable outcomes in seizure control, even in cases of drug-resistant epilepsy. Successful microsurgical AVM resection immediately eliminates the malformation and demonstrates the highest obliteration rates compared to other treatment modalities [16]. Complete AVM obliteration is the primary therapeutic objective, and expert-led case series have reported success rates of approximately 95% [17]. Accelerated treatment is particularly crucial in the setting of AVM rupture, as it is associated with an increased risk of bleeding within the first year [18]. Nevertheless, microsurgical AVM excision may require a prolonged dissection process, meticulous verification of all afferent and efferent vessels, and careful navigation of surrounding vascular structures [19].

Our findings support previous evidence demonstrating the efficacy of microsurgical resection in achieving seizure control in AVM-related epilepsy. In our cohort, a statistically significant association was observed between seizure duration and postoperative outcomes, with prolonged seizure history correlating with poorer Engel and ILAE scores (*p* = 0.005, Table 3 and Table 4). This aligns with the results from von der Brelie et al., who similarly found that longer symptom duration in drug-resistant epilepsy (DRE) patients was associated with worse seizure outcomes following surgery [20]. Furthermore, our observed seizure-free rate of 78% is consistent with seizure-free rates reported in larger series, which range from 77% to 91% following microsurgical AVM removal [21].

The significance of early surgical intervention is further reinforced by the pathophysiological mechanisms discussed in the recent literature. Choque-Velasquez et al. [21] described how chronic hypoperfusion and gliosis in perinidal tissue—driven by the “steal phenomenon”—create a hyperexcitable cortex conducive to epileptogenesis. Removing the AVM may disrupt this cycle by restoring hemodynamic balance and eliminating the epileptogenic substrate. In light of this, early surgical resection in selected patients with treatment-refractory epilepsy could not only prevent hemorrhagic complications but also enhance long-term seizure control outcomes [21].

Possible mechanisms underlying seizure development in AVM patients include structural and functional changes in the surrounding brain parenchyma. Chronic hypoxia, gliosis, and hemosiderin deposition from prior hemorrhage or micro-bleeds may increase cortical excitability and predispose patients to seizures. Possible mechanisms of seizure remission include removal of the epileptogenic nidus, reduction in perilesional gliosis, decreased cortical irritation, and the restoration of hemodynamic balance. Chronic hypoxia, hemosiderin deposition, and gliotic scarring may contribute to epileptogenesis, and their removal may restore network homeostasis [22]. Additionally, the hemodynamic effects of high-flow shunting through AVMs can lead to perilesional ischemia, further contributing to epileptogenesis [23].

Arteriovenous malformations exhibit unique clinical presentations, and the success of surgical treatment largely depends on appropriate patient selection, which is best achieved in specialized neurosurgical centers. The Spetzler–Martin grading system is used to determine the optimal treatment approach for AVMs. For low-grade AVMs with a low risk of complications, treatment options are generally successful and commonly employed. Conversely, for high-grade AVMs, observation and symptomatic management are typically recommended, with a careful assessment of the risk–benefit ratio before any intervention. Unfavorable surgical outcomes are also associated with the patient’s age and the presence of hemorrhage. Inadequate surgical resection can lead to recurrent bleeding and damage to surrounding structures. According to the literature, the risk of disability and mortality during AVM removal ranges from 2 to 8%. For grade IV and V AVMs, the surgical risk is very high, suggesting the need for an integrated approach, including endovascular therapy or radiosurgery [11,23,24].

Recent research has shown that radiological or endovascular approaches can reduce the AVM nidus, enabling surgical intervention to be considered at a later stage, if appropriate. Our series of observations affirm the safety and efficacy of this proposed multimodal treatment strategy, which aligns with international standards. Alongside surgical proficiency, thoughtful decision making and meticulous patient selection are essential for the successful management of patients with arteriovenous malformations [25].

Seizure outcomes vary across treatment modalities, with microsurgery demonstrating the highest rates of seizure freedom due to immediate and complete AVM removal. Endovascular embolization can reduce AVM size and flow, serving as an adjunctive therapy that may help in seizure control, particularly in cases of partial AVM obliteration. Radiosurgery offers a non-invasive option with gradual nidus obliteration over months to years; however, its effects on seizures are less immediate, and seizure control is often achieved only after complete nidus closure [26].

Kazakhstan has had access to Gamma Knife radiosurgery since 2021, further expanding the treatment options available for AVM patients. This advancement allows for a comprehensive multimodal approach tailored to individual cases, emphasizing the importance of specialized centers equipped with diverse therapeutic modalities.

### Limitations

This study has several limitations. As a retrospective, single-center analysis, it is subject to inherent selection and information biases. Although the National Center for Neurosurgery serves as the primary referral institution for AVM treatment in Kazakhstan, a portion of patients was lost to follow-up, which may influence the accuracy of long-term seizure outcome data. From an initial cohort of 421 AVM cases, 116 patients presented with seizures, yet only 27 met the full inclusion criteria, limiting the sample size and, consequently, the generalizability of the findings. While Gamma Knife radiosurgery became available in Kazakhstan in 2021, the limited follow-up duration of patients treated with this modality prevents a meaningful assessment of its long-term efficacy in seizure control. Furthermore, the absence of a multicenter approach restricts the broader applicability of the results. Despite these limitations, this study demonstrated a 78% seizure-free rate following microsurgical resection, with favorable trends observed across different AVM grades. These findings suggest promising outcomes for carefully selected patients. Nonetheless, prospective, multicenter studies with larger cohorts and extended follow-up are warranted to validate these results and refine treatment strategies for AVM-associated epilepsy.

## 6. Conclusions

The findings of this study suggest that seizures are commonly observed in patients with cerebral arteriovenous malformations, particularly in those with a history of hemorrhage, which can significantly impact the patient’s quality of life. Furthermore, seizures are more frequently associated with large AVMs located in the frontotemporal regions among patients without a history of hemorrhage, highlighting the significance of meticulous patient selection. The results of the present investigation indicate that the majority of patients experienced a reduction in seizure frequency following microsurgical resection of the AVM. Careful preoperative planning and an integrated multimodal treatment approach, including endovascular and surgical interventions, are key to achieving favorable outcomes in the management of patients with AVMs and symptomatic epilepsy.

Future multicenter studies are warranted to identify predictors of seizure remission and to optimize treatment strategies combining microsurgery with embolization or radiosurgery.

## Figures and Tables

**Table 1:** Baseline characteristics.

Baseline Characteristics of the Patient Cohort		
Gender		
Male	14	52%
Female	13	48%
Seizure character		
Focal	11	41%
Generalized	16	59%
Presence of aura		
Yes	11	41%
No	16	59%
Presurgical embolization		
Total	15	55.5%
Once	12	44.4%
Three-times	3	11.1%

**Table 2:** Seizure outcomes.

ENGEL Classification		
IA	17	62.96%
IB	3	11.1%
IIA	2	7.41%
IIIA	3	11.1%
IVB	2	7.41%
ILAE classification		
1	16	59%
3	8	29.9%
5	3	11.1%

**Table 3:** Years of seizure and postsurgical seizure outcome by ENGEL classification.

		ENGEL Outcomes by Groups			Total
		Positive	Moderate	Negative	
Years with seizures	<5 years	11	1	0	12
	5–10 years	4	3	0	7
	>10 years	3	1	4	8
Total		18	5	4	27
					***p*** **-Value 0.005**

**Table 4:** Years of seizure and postsurgical seizure outcome by ILAE classification.

		ILAE Class			Total
		1	3	5	
Years with seizures	<5 years	10	2	0	12
	5–10 years	4	3	0	7
	>10 years	2	3	3	8
Total		16	8	3	27
					***p*** **-Value 0.03**

**Table 5:** Antiseizure medications’ characteristics.

Antiseizure Medication Usage		
	Before	After the Operation
Yes	17	14
No	10	13
The name of antiseizure medication		
	Before	After the operation
Finlepsin	6	1
Valproic acid	3	3
Carbamazepine	8	10

## Data Availability

All data are available upon request.
